# Microsatellite variation and genetic structure of brook trout (*Salvelinus fontinalis*) populations in Labrador and neighboring Atlantic Canada: evidence for ongoing gene flow and dual routes of post-Wisconsinan colonization

**DOI:** 10.1002/ece3.200

**Published:** 2012-05

**Authors:** Brettney L Pilgrim, Robert C Perry, Donald G Keefe, Elizabeth A Perry, H Dawn Marshall

**Affiliations:** 1 Department of Biology, Memorial University of Newfoundland and LabradorSt. John's, Newfoundland and Labrador, A1B 3X9, Canada; 2 Department of Environment and Conservation, Wildlife Division, Government of Newfoundland and LabradorCorner Brook, Newfoundland and Labrador, A2H 7S1, Canada; 3 Genomics and Proteomics Facility, CREAIT Network, Memorial University of Newfoundland and LabradorSt. John's, Newfoundland and Labrador, A1B 3X9, Canada

**Keywords:** Brook trout, glacial refugia, microsatellites, population structure, postglacial colonization, *Salvelinus fontinalis*

## Abstract

In conservation genetics and management, it is important to understand the contribution of historical and contemporary processes to geographic patterns of genetic structure in order to characterize and preserve diversity. As part of a 10-year monitoring program by the Government of Newfoundland and Labrador, Canada, we measured the population genetic structure of the world's most northern native populations of brook trout (*Salvelinus fontinalis*) in Labrador to gather baseline data to facilitate monitoring of future impacts of the recently opened Trans-Labrador Highway. Six-locus microsatellite profiles were obtained from 1130 fish representing 32 populations from six local regions. Genetic diversity in brook trout populations in Labrador (average *H*_E_= 0.620) is within the spectrum of variability found in other brook trout across their northeastern range, with limited ongoing gene flow occurring between populations (average pairwise *F*_ST_= 0.139). Evidence for some contribution of historical processes shaping genetic structure was inferred from an isolation-by-distance analysis, while dual routes of post-Wisconsinan recolonization were indicated by STRUCTURE analysis: *K*= 2 was the most likely number of genetic groups, revealing a separation between northern and west-central Labrador from all remaining populations. Our results represent the first data from the nuclear genome of brook trout in Labrador and emphasize the usefulness of microsatellite data for revealing the extent to which genetic structure is shaped by both historical and contemporary processes.

## Introduction

The extent and structure of genetic variation across a species’ range are informative about both historical processes, such as the pattern of recolonization after previous glacial maxima (Avise 2000), and contemporary ones, including ongoing dispersal and gene flow among populations. From a conservation and management perspective, it is important to understand the contribution of each type of process to the observed geographic pattern of genetic structure in order to identify evolutionarily distinct lineages and maximally preserve diversity. Effective management requires the maintenance of genetic diversity to sustain a population's ability to evolve (Frankham et al. 2002) and persevere through environmental changes (Moritz and Faith 1998; Avise 2004), such as climate change, biological invasions, or anthropogenic threats like pollution, habitat fragmentation, or over-exploitation.

**Figure 1:**
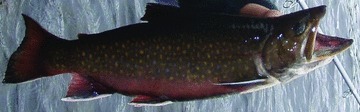
The study species, brook trout (*Salvelinus fontinalis*). Photograph by Donald Keefe.

Brook trout (*Salvelinus fontinalis* Mitchill, 1814; Fig. 1) are primarily freshwater salmonid fish (Scott and Crossman 1973) that inhabit the clear, cool waters of streams, rivers, and lakes (Behnke 1972). Coastal populations with ocean access are sometimes characterized by anadromous forms of this species (Power 1980), contributing to its ecological complexity. Due to their economic value as game fish, brook trout have been widely introduced to temperate regions outside their native range and now exist as numerous naturalized populations. Endemic to eastern North America (McCrimmon and Campbell 1969), the native range of the species includes much of eastern Canada, and extends southward to the northern tip of Georgia. Labrador, the mainland component of the province of Newfoundland and Labrador in the Atlantic region of Canada, constitutes the northeasternmost portion of the native range. Labrador comprises the easternmost part of Canadian Shield and consists of two climate types, polar tundra in the north and subarctic in the south.

In Labrador, brook trout are the most important game fish (DFO 2007), and are under increased anthropogenic threat due to the recent opening of the Trans-Labrador Highway (TLH). This threat consists of two parts. First, culvert placement along the expanse of the highway has been poor (Gibson et al. 2005), and the habitat fragmentation that the highway imposes could interfere with migration, thus reducing gene flow and subdividing current populations into smaller isolated populations. Previous studies have reported the loss of brook trout from much of their native range (50% of brook trout have disappeared from their historical habitat in Connecticut alone) (Hudy et al. 2008) likely due to land development and concomitant habitat alteration (Stranko et al. 2008; Kanno et al. 2010; Waco and Taylor 2010). Second, fishing pressure may increase as lakes become more easily accessible; previously, the majority of the lakes in Labrador were accessible by float plane only. The Newfoundland and Labrador Department of Environment and Conservation has established a 10-year monitoring program of brook trout populations in proximity of the newly developed highway in Labrador. Assessment of the current population genetic structure throughout Labrador is crucial in order to lay the groundwork for future temporal samples of the same populations for monitoring of population trends.

Several studies have established the role of contemporary factors in shaping the genetic structure of brook trout populations across portions of the species’ native range, including Maine (Castric et al. 2001), Québec (Angers et al. 1995, 1999; Angers and Bernatchez 1998; Fraser et al. 2004), New Brunswick (Rogers and Curry 2004), and insular Newfoundland (Adams and Hutchings 2003). Ecological variables were found to affect the patterning of genetic structure in certain cases. For example, in Miramichi River, New Brunswick, population structure was dictated by habitat selection and the life history strategy of anadromy (Rogers and Curry 2004), while the structure of brook trout populations in Mistassini Lake, Québec, was attributed to dispersal patterns, specifically male-biased dispersal among tributaries and female-biased dispersal from tributaries to outflow populations (Fraser et al. 2004). Another major factor that shapes the population genetic structure of freshwater fish such as brook trout is the structure of the habitat. In particular, the connectivity of lakes within watersheds has been found to influence the pattern of genetic diversity of brook trout on a microgeographic scale (Angers et al. 1995, 1999; Angers and Bernatchez 1998; Castric et al. 2001; Adams and Hutchings 2003).

A species’ genetic structure is also strongly influenced by historical events like the Wisconsinan glaciation which reached its maximum 18,000 years ago. Relative to recently reestablished northern populations, populations that persisted in isolation in southern refugia have had time to accumulate variation (Hewitt 1996, 1999; Bennett 1997) while simultaneously diverging from populations in other refugia (Hewitt 1996, 2004). This signature is reflected in the distribution and genetic structure of populations today – northern regions typically have fewer species (Pielou 1991) characterized by lower genetic diversity, a pattern that is referred to by Hewitt (1996) as “southern richness, northern purity.” The pattern of population genetic structure therefore reveals the direction of colonization and the contribution of distinct refugial origins to the present-day species’ distribution.

The pattern of colonization of brook trout throughout their native range was assessed by Danzmann et al. (1998) on the basis of mtDNA diversity. Brook trout in eastern Canada were shown to possess very little genetic variation relative to the rest of the range, comprising only 13 haplotypes of a possible 61. This part of the range was primarily colonized by haplotype “1,” postulated to have been present in both the Mississippian and Atlantic refugia. However, the presence of 10 private haplotypes combined with the lack of haplotype “2” fish suggests that fish in this region may instead have originated from a more northeasterly refugium (Danzmann et al. 1998), also referred to as the Acadian refugium (Schmidt 1986). Although Danzmann et al. (1998) did not consider Labrador brook trout, Black et al. (1986) inferred from species distributional patterns that brook trout recolonized inland Labrador via Québec from a combination of the Mississippian and Atlantic refugia while simultaneously recolonizing coastal regions of Labrador from the Atlantic refugium. This pattern is inconsistent with Danzmann et al. (1998).

As part of the Department of Environment and Conservation's monitoring program, we sought to determine the genetic structure of brook trout populations in Labrador as recorded by nuclear microsatellite loci. Such analyses will provide the baseline information about current population structure needed to monitor population trends impacted by the TLH. In addition, we test whether patterns of genetic variation are influenced more strongly by historical or contemporary events. Since Labrador brook trout populations are at their northern range extreme, we hypothesize that they maintain the genetic signature of postglacial recolonization. Since the mtDNA phylogeography and distributional patterns of fishes in Labrador did not clearly elucidate postglacial colonization routes of brook trout, we also investigated the number and source of refugial populations that colonized Labrador.

## Methods and Materials

### Sample collection

**Figure 2:**
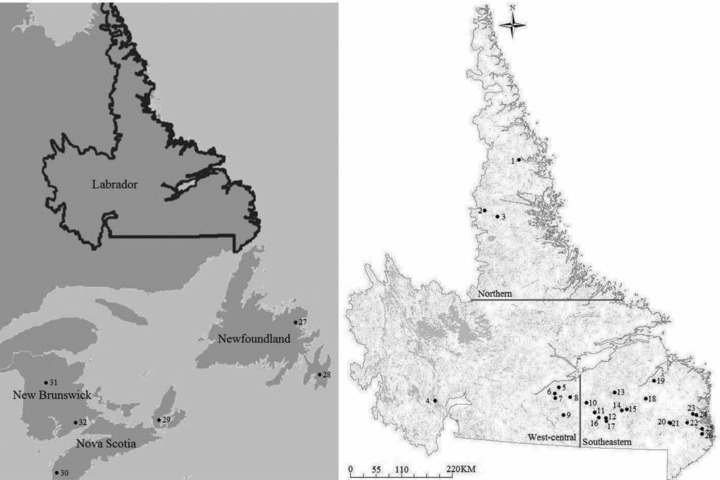
Lakes sampled for brook trout across eastern Canada. Population codes are given in Table 2.

Brook trout caudal fin clippings from fish in 31 lakes within 18 watersheds from six regions across eastern Canada (northern, west-central and southeastern Labrador, insular Newfoundland, Nova Scotia, and New Brunswick; Fig. 2) were sampled via gill netting by provincial wildlife biologists from 2003 to 2008 (1094 samples). Fin clippings were stored at –20°C. DNA extractions of fish from Atikonak Lake in west-central Labrador (36) were provided by Dr. Steven M. Carr (Memorial University of Newfoundland and Labrador).

### Microsatellite analysis

Genomic DNA was extracted from ∼25 mm^2^ of the caudal fin clipping using the Qiagen QIAmp DNA Mini Kit (Qiagen Inc., Mississauga, Canada) following the tissue protocol. Nine microsatellite loci were initially tested: *Sfo*12, *Sfo*18 (Angers et al. 1995), *MST*85 (Presa and Guyomard 1996), *Sfo*D91, *Sfo*B52, *Sfo*C129, *Sfo*D100, *Sfo*C86, *Sfo*D75 (T.L. King, US Geological Survey, unpubl. data). PCRs were carried out in an Applied Biosystems GeneAmp PCR System 9700 Thermal Cycler (Applied Biosystems Inc., Carlsbad, CA) and contained 1X PCR Master Mix (Promega Corp., Madison, WI), 2 µL 10 mM of each primer and 2 µL genomic DNA (2–570 ng). Four fluorescent dyes were used to label forward primers as follows: PET –*Sfo*12, *SfoD*100; VIC –*Sfo*18, *MST*85, and *SfoC*129; 6-FAM –*SfoD*91, *SfoB*52, and *SfoC*86; NED –*SfoD*75. For primer pairs *Sfo12, Sfo18,* and *MST85* the PCR profile was as follows: 95°C for 4 min; 35 cycles of 95°C for 1 min, 55°C for 45 s, and 72°C for 45 s; 72°C for 7 min. For the remaining loci the PCR profile was: 95°C for 4 min; 35 cycles of 94°C for 45 s, 56°C for 45 s and 72°C for 1.5 min; 72°C for 5 min. Prior to electrophoresis, reactions were diluted (1:4), and 1 µL was added to 8.8 µL of formamide and 0.2 µL of LIZ-500 size standard. Loci were multiplexed as follows: (1) *Sfo*12, *Sfo*18, *SfoD*91; (2) *Sfo*B52, *Sfo*C129, *Sfo*D100; (3) *Sfo*C86, *Sfo*D75, *MST*85. Samples were electrophoresed in an Applied Biosystems 3730 DNA Analyzer using GeneScan Software (Applied Biosystems Inc., Carlsbad, CA). The six loci with the greatest amplification success rate (*Sfo*18, *Sfo*B52, *Sfo*C129, *Sfo*D100, *Sfo*C86, and *Sfo*D75) were selected for subsequent analysis.

### Data analysis

Diversity at each locus was quantified by calculating observed (*H*_O_) and expected (*H*_E_) heterozygosity and number of alleles (*N*_A_) in FSTAT 2.9.3 (Goudet 1995). The inbreeding coefficient (*F*_IS_) at each locus was estimated using the Weir and Cockerham (1984) algorithm as implemented in FSTAT (120 randomizations). Linkage disequilibrium among all pairs of loci was evaluated with the log likelihood ratio test (G-test) of GENEPOP 1.2 (Raymond and Rousset 1995) using the default Markov chain parameters. Diversity measures (*N*_A_, *H*_O_, *H*_E_) and allelic richness (*A*) were calculated in FSTAT for each population (defined as all fish sampled in a lake), as well as each region (defined by Anderson [1985] according to geology, vegetation, and fish distributions, with watersheds in each region draining to a specific inlet or region of coast). The rank-based Kruskal–Wallis test was used to determine if differences between overall *H*_E_ and *A* of each lake were significant. *F*IS values for each locus in each lake were calculated in FSTAT.

To determine whether mutation as well as drift has influenced differentiation, we used the allele size randomization test (following Hardy et al. 2003) of SPAGeDi 1.3 (Hardy and Vekemans 2002). Randomly permuting alleles (2000 iterations) provides a 95% confidence interval of the simulated distribution of *R*_ST_ values (or ρRST). If *R*_ST_ > *F*_ST_, *R*_ST_ will fall outside of the ρ*R*_ST_ 95% confidence interval, and differentiation has been influenced by mutation. If it falls within this range, *R*_ST_ does not significantly differ from *F*_ST_, indicating that mainly drift contributes to differentiation. Since we found that *R*_ST_ (0.153) was not significantly different from *F*_ST_ (*F*_ST_= 0.144; *P*= 0.115) (Table 3), pairwise *F*_ST_ values, as estimated in ARLEQUIN 3.1 (Excoffier et al. 2005), were used in subsequent analyses. Due to greater variance *R*_ST_ is less reliable at detecting population differentiation than *F*_ST_ (Sefc et al. 2007).

To assess the partitioning of genetic diversity among populations, an analysis of molecular variance (AMOVA) was performed in ARLEQUIN 3.1 (Excoffier et al. 2005), with three hierarchical levels to quantify the variance among (1) lakes within regions; (2) watersheds within regions; and (3) lakes within watersheds. In addition, Nei et al.'s (1983)*D*_A_ distance (calculated with MICROSATELLITE ANALYSER 4.05 [Dieringer and Schlötterer 2003]) was used to assess distance-based relationships among watersheds. The neighbor-joining dendrogram was generated using the NEIGHBOUR and CONSENSE modules in PHYLIP 3.69 (Felsenstein 1989), and visualized in TREEVIEW 1.4 (Page 1996).

To characterize the correlation of genetic distances and geographical distances, we conducted an isolation-by-distance analysis. Pairwise estimates of *F*_ST_/(1–*F*_ST_) were regressed against geographical distance (Rousset 1997) using the ISOLDE program within GENEPOP. Distances were measured in two ways: (1) in a straight line overland between lakes, and (2) between lakes along extant waterways, to determine if historical or contemporary influences play the more significant role in shaping observed genetic structure. Distances were measured using ESRI ArcView 9 (Redlands, CA).

STRUCTURE 2.1 (Pritchard et al. 2000) was used to identify the number of genetically distinct clusters (*K*) across all sampled lakes. First we wanted to discern between the possible routes of postglacial recolonization, either from a combination of Atlantic and Mississippian refugia, or from the Acadian refugium. Secondly we wanted to evaluate the overlay of contemporary patterns of gene flow. For each value of *K*, five iterations were run to assess convergence of the likelihood with a burn-in period of 50,000, followed by 200,000 iterations for values of *K*= 1 through 20. Each simulation was performed using an ancestry model incorporating admixture and correlated allele frequencies, without prior population information (Falush et al. 2003). *K* was selected in two ways: (1) as the highest value of ln Pr(X|*K*), as recommended by Pritchard et al. (2000) and (2) according to an ad hoc statistic described by Evanno et al. (2005).

## Results

### Per-locus diversity and linkage disequilibrium

A total of 1130 brook trout from 32 lakes within 19 watersheds across six regions of eastern Canada were genotyped at six microsatellite loci. An average of 17 alleles (*N*_A_) across all six loci was found, with a range of eight alleles at *Sfo*C129 to 31 alleles at *Sfo*D91 (Table 1). Observed heterozygosity (*H*_O_) ranged from 0.528 (*Sfo*C129) to 0.807 (*Sfo*D100), with an average of 0.642. A preliminary indication of genetic structure was detected by deviations from Hardy–Weinberg equilibrium. *F*_IS_ was significantly greater than zero for four loci (*Sfo*18, *Sfo*B52, *Sfo*D75, *Sfo*D91) and overall after correction for multiple comparisons (*P*= 0) (Table 1). Significant linkage disequilibrium after Bonferroni correction was found for only three of 480 pairwise comparisons of loci by the log likelihood ratio test (G-test) of GENEPOP (*P* < 0.0001).

**Table 1:** Allelic variation at each microsatellite locus. Number of alleles (*N*_A_), observed (*H*_O_) and expected (*H*_E_) heterozygosity and *F*_IS_ (Wright's inbreeding coefficient). Significant heterozygote excess/deficit at *P* < 0.05 after Bonferroni correction (α= 0.00833) denoted with an asterisk.

Locus	*H*_O_	*H*_E_	*N*_A_	*F*_IS_
*Sfo*18	0.757	0.597	19	0.211^*^
*Sfo*B52	0.643	0.576	15	0.105^*^
*Sfo*C129	0.527	0.528	8	–0.001
*Sfo*D75	0.698	0.616	17	0.118^*^
*Sfo*D91	0.872	0.732	31	0.161^*^
*Sfo*D100	0.779	0.807	12	–0.036
Weighted averages	0.717	0.642	17	0.098^*^

### Within population variation

Levels of genetic variation were highest in Farnham Brook in Nova Scotia (*H*_E_= 0.727, *A*= 4.910), and lowest in Dead Dog Pond in central Labrador (*H*_E_= 0.370, *A*= 2.616) (Table 2). When populations were assigned to their corresponding region, genetic diversity was highest in Newfoundland in terms of expected heterozygosity (*H*_E_= 0.702) and in New Brunswick based on allelic richness (*A*= 8.347) and lowest in northern Labrador (*H*_E_= 0.682, *A*= 6.272) (Table 2). The Kruskal–Wallis analysis of variance showed no significant difference between average expected heterozygosity (over all loci) and allelic richness on either a per-lake or a regional basis (*P* > 0.05). However, there was a significant negative correlation between allelic richness and latitude (*R*^2^= 0.214; *P*= 0.009), as well as a significant positive correlation between heterozygosity and longitude in brook trout populations found in west-central and southeastern Labrador (*R*^2^= 0.2476; *P*= 0.0184), after outliers were removed. No significant deviations from Hardy–Weinberg equilibrium were observed per locus among lakes or overall (*P* > 0.05).

**Table 2:** Locations with population code, sample sizes (*N)*, observed (*H*_O_) and expected (*H*_E_) heterozygosity, and allelic richness (*A,* corrected to n = 7) of each lake and across each region.

Region	Watershed	Lake	*N*	*H*_E_	*H*_O_	*A*
**Northern Labrador**			36	0.654	0.682	6.272
1	Saputit Brook	Saputit Lake	7	0.714	0.658	4.000
2	Kogaluk River	Cabot Lake	16	0.683	0.609	3.329
3	Iladlivik Brook	Walkabout Lake	13	0.585	0.639	3.715
**West-central Labrador**			207	0.585	0.700	6.985
4	Atikonak Lake	Atikonak River	22	0.574	0.649	4.245
5	Traverspine River	No Boat Pond	45	0.385	0.470	3.378
6	Traverspine River	The Right Lake	17	0.627	0.608	4.013
7	Kenamu River	Mercier Lake	15	0.491	0.616	3.739
8	Kenamu River	Brennan Lake	58	0.722	0.694	4.265
9	Kenamu River	Nikki's Pond	50	0.623	0.588	3.635
**Southeastern Labrador**			734	0.650	0.692	8.118
10	Eagle River	Fred's Lake	53	0.727	0.691	4.491
11	Eagle River	No Name Lake	27	0.595	0.616	3.935
12	Eagle River	Nap Pond	33	0.613	0.630	4.238
13	Eagle River	Nippard's Lake	60	0.584	0.568	3.348
14	Eagle River	Osprey Lake	37	0.653	0.610	3.542
15	Eagle River	Dead Dog Pond	11	0.268	0.370	2.616
16	St. Augustine River	St. Augustine	54	0.586	0.603	3.776
17	St. Augustine River	Bog Lake	59	0.558	0.574	3.633
18	Paradise River	Keith's Lake	41	0.603	0.591	4.072
19	Paradise River	Crooked Lake	55	0.521	0.591	4.177
20	Alexis River	Alexis Pond	45	0.806	0.667	4.056
21	Alexis River	Handkerchief Pond	29	0.673	0.575	3.839
22	Alexis River	Feeder Pond	24	0.627	0.603	4.004
23	Gilbert River	Tilt Pond	46	0.706	0.635	4.450
24	Gilbert River	Gilbert Lake	60	0.672	0.592	4.048
25	St. Lewis River	Curl's Pond	61	0.817	0.688	4.457
26	St. Mary's River	Mary's Harbour Big Pond	39	0.719	0.680	4.632
**Newfoundland**			54	0.654	0.702	6.671
27	Middle Brook	Butt's Pond	25	0.639	0.637	4.330
28	Salmonier River	Little Gull Pond	29	0.662	0.628	3.590
**Nova Scotia**			50	0.690	0.694	7.889
29	River Denys	Alder Brook	30	0.643	0.620	4.391
30	Salmon River	Farnham Brook	20	0.769	0.727	4.910
**New Brunswick**			49	0.706	0.690	8.347
31	Miramichi River	Moose Lake	25	0.647	0.649	4.641
32	Inner Bay of Fundy	Walton Lake	24	0.767	0.663	4.159

**Table 3:** Summary of allele size permutation test as implemented in SPAGeDi showing estimates of *R*_ST_, simulated *R*_ST_ (ρ*R*_ST_) values and their 95% confidence interval and *F*_ST_ following 2000 allele permutations.

Locus	*R*_ST_	ρ*R*_ST_	95% CI	*F*_ST_	*P*
*Sfo*18	0.166	0.127	0.045–0.267	0.156	0.297
*Sfo*B52	0.131	0.114	0.054–0.204	0.130	0.193
*Sfo*C129	0.145	0.150	0.084–0.200	0.167	0.709
*Sfo*D75	0.137	0.152	0.062–0.267	0.185	0.837
*Sfo*D91	0.112	0.076	0.045–0.166	0.089	0.100
*Sfo*D100	0.128	0.088	0.044–0.169	0.101	0.169
All	0.153	0.130	0.087–0.176	0.144	0.115

### Population genetic structure

The mean *F*_ST_ measured among pairs of lakes was 0.139 and comparisons were significantly (*P* < 0.05) positive except those involving Dead Dog Pond (which had the smallest sample size of *N*= 11). The significant pairwise *F*_ST_ measures ranged from 0.014 between Gilbert Lake and Feeder Pond (both in southeastern Labrador), to 0.374 between Atikonak Lake (west-central Labrador) and Alder Brook (Nova Scotia). The average pairwise *F*_ST_ among lakes in a watershed measured 0.070. The isolation-by-distance analysis among all populations revealed a significant correlation between *F*_ST_/(1–*F*_ST_) and both overland and current waterway distances, but was more highly significant for overland distances (*P*= 0 vs. *P*= 0.002).

Both AMOVA analyses detected significant geographic structuring within and among populations and regions (*P* < 0.05; Table 4). The first analysis defined groups by lakes, then watersheds. Most of the variation was detected within lakes, followed by among watersheds, then among lakes within watersheds. In the second analysis, fish were grouped by watershed, then into regions. Most of the variation was detected within watersheds, followed by among watersheds within regions, and the least was among regions. Overall, the majority of variation is within lakes, and the next most significant factor explaining variation is watershed. There were very low but significant levels of differentiation (*P*= 0.03) among regions.

**Table 4:** Hierarchical analysis of molecular variance in brook trout populations across northeastern Canada performed by grouping fish into populations (either by lake or watershed), then subsequently into either watersheds or regions. Percentage of total variance (%) and *F*-statistics (*F*_CT_, *F*_SC_, *F*_ST_) for each hierarchical level represented.

	Among regions/watersheds		Among populations within watersheds/regions		Within populations	
Comparison	%	*F*_CT_	%	*F*_SC_	%	*F*_ST_
i. Lakes grouped into watersheds	7.516	0.075	6.550	0.071	85.935	0.141
ii. Watersheds grouped regionally	1.313	0.013	10.455	0.106	88.232	0.118

**Figure 3:**
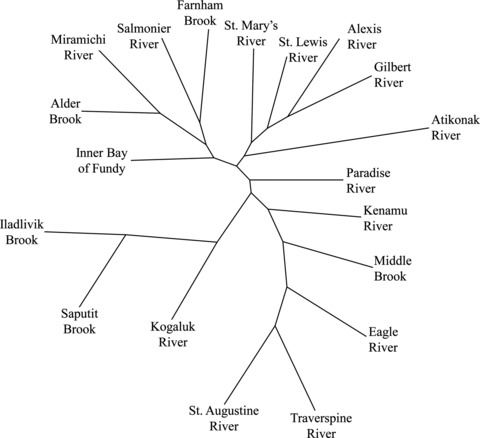
Unrooted neighbor-joining denrogram using Nei et al.'s (*D*_A_) distance (1983) relating the 33 brook trout populations sampled across eastern Canada.

Genetic structure was also investigated using Nei's genetic distance (*D*_A_) (Nei et al. 1983), the most appropriate distance measure when drift is the main force contributing to differentiation (Paetkau et al. 1997). Following the AMOVA results, fish were grouped into watersheds for this analysis. The neighbor-joining dendrogram of *D*_A_ distances illustrates overall low genetic differentiation among watersheds with some regional pattern (Fig. 3). All watersheds in northern Labrador constitute one cluster. Watersheds from New Brunswick, Nova Scotia, and Newfoundland, with the exception of Middle Brook, cluster together. All watersheds in southeastern Labrador that drain directly into the Atlantic Ocean (south of Sandwich Bay) cluster together. Watersheds in southeastern and west-central Labrador that drain from Sandwich Bay and Lake Melville, respectively, cluster together.

**Figure 4:**
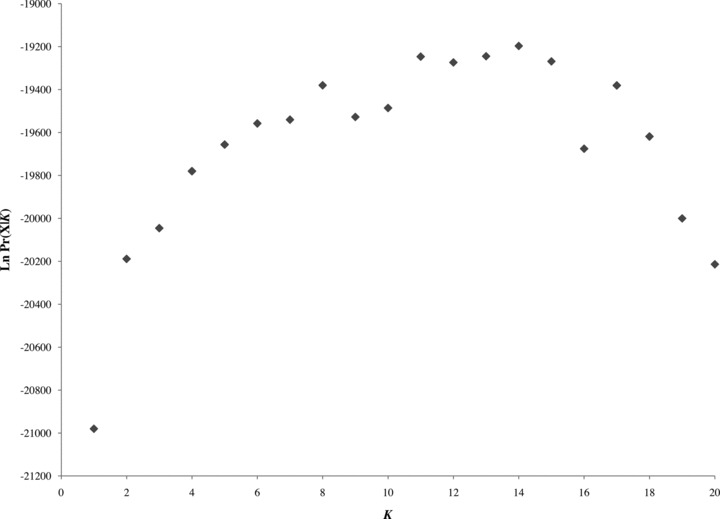
A plot of the log likelihood of the data (ln Pr(X|*K*)) values obtained from the STRUCTURE analysis for *K*= 1–15.

**Figure 5:**
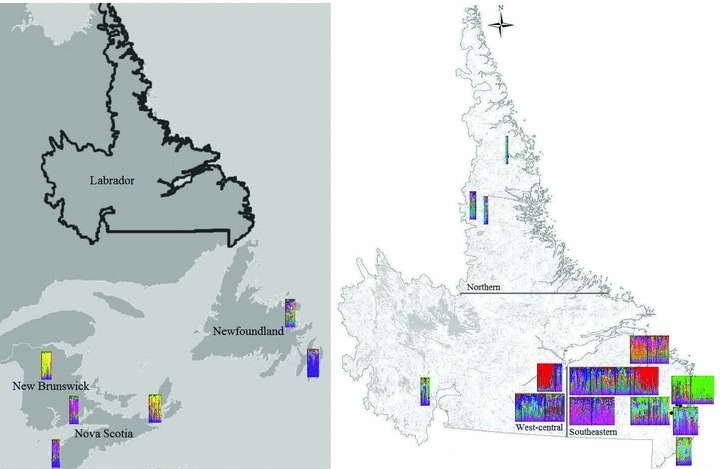

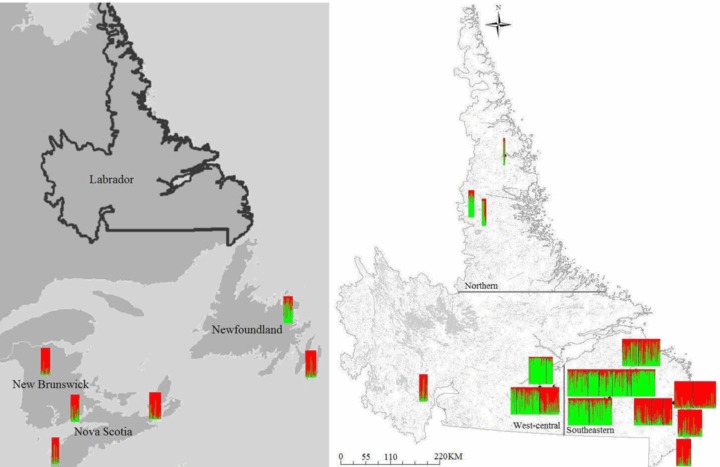
Geographical distribution of genetic clusters inferred from the STRUCTURE analysis based on *K*= 14 (A) and *K*= 2 (B) for each watershed sampled. Each bar represents an individual fish with each color representing a genetic group or cluster. Bar charts are sized according to sample size, and lakes within the same watershed appear together.

From the STRUCTURE analysis, under the Pritchard et al. (2000) criterion, the maximum value of ln Pr(X|*K*) (–19197.2) corresponds to *K*= 14 (Fig. 4). Upon mapping the bar plots of individual estimated cluster membership coefficients of *K*= 14 (Fig. 5A), lakes within the same watershed tend to have similar cluster assignments throughout west-central and southeastern Labrador. Exceptions to this pattern include Nikki's Pond (Kenamu River), which has a cluster assignment distinct from any other lake or watershed; The Right Lake (Traverspine River), which clusters with Eagle River; Nippard's Lake (Eagle River); and No Boat Pond (Traverspine River), which have similar cluster assignments distinct from their source watersheds. We note that one cluster assignment dominates all three watersheds in northern Labrador, and the two watersheds in Newfoundland have distinct cluster assignments from each other and all other watersheds, while Nova Scotia and New Brunswick are characterized by a heterogeneous cluster assignment distinct from the other regions.

Under the ad hoc criterion of Evanno et al. (2005)*K*= 2. Evanno et al. (2005) recommended this statistic when ln Pr(X|*K*) values plateau once the optimal *K* has been reached, as was the case here (Fig. 4). Bar plots of estimated individual cluster membership coefficients for *K*= 2 are mapped in Figure 5B. The two clusters consist of (1) Northern Labrador and all populations (except Atikonak Lake) that drain into Lake Melville and all populations that drain into Sandwich Bay and (2) the coastal populations of southeastern Labrador, insular Newfoundland, Nova Scotia and New Brunswick, and Atikonak Lake.

## Discussion

We investigated population genetic structure of brook trout in Labrador and the broader region of northeastern North America, including insular Newfoundland, Nova Scotia, and New Brunswick, by measuring variation at six microsatellite loci. We have collected the first genetic data from the nuclear genome on brook trout in Labrador, providing us with baseline data about current population structure needed to monitor future impacts of the recently opened TLH. We also make inferences about patterns of post-Wisconsinan colonization of Labrador by brook trout, which are significant in the broader context of postglacial recolonization and the biogeography of fishes in Atlantic Canada.

### Patterns of intrapopulation genetic variability reflect putative postglacial recolonization routes

Levels of expected heterozygosity in brook trout populations in Labrador, Canada (average *H*_E_= 0.620), are within the spectrum of variability observed throughout the northeastern range (*H*_E_= 0.380–0.778 [Angers and Bernatchez 1998; Adams and Hutchings 2003; Rogers and Curry 2004; Poissant et al. 2005]). Since Labrador is located at the northernmost edge of the brook trout's native range, diversity was expected to be reduced due to relatively recent bottlenecks and founder events. However the range of *H*_E_ reported in other studies is so broad and considers so many different geographic scales that it is difficult to directly accept or reject this hypothesis. However, the comparatively moderate levels of microsatellite genetic diversity we report is inconsistent with the only other study to investigate genetic variation in brook trout populations in Labrador. In an assessment of the intraspecific population genetic structure of Atikonak Lake in western Labrador (S. Carr, unpubl. report) only one mtDNA haplotype (401 bp of the cytochrome b gene) was found among 23 individuals. Similarly a wide-scale survey of brook trout RFLP variation showed that 98% of the fish that colonized northeastern North America were of a single haplotype (Danzmann et al. 1998). Clearly, the rapidly evolving nuclear microsatellite loci are potentially more informative than mtDNA for investigating population structure in this recently colonized region.

As measured by allelic richness, brook trout in northeastern Canada demonstrate the characteristic “southern richness, northern purity” (Hewitt 1996) pattern; *A* is highest in Nova Scotia and lowest in northern Labrador (Table 1). In fact there is a significantly negative correlation between allelic richness and latitude (*P*= 0.018); this supports a route of postglacial recolonization through northeastern Canada from more southern refugia, resulting in fewer alleles in northern populations due to a bottleneck effect.

The negative correlation between expected heterozygosity and latitude was not significant (*P* > 0.05), probably because *H*_E_ is lowest overall in west-central rather than northern Labrador. Part of west-central Labrador is elevated on a plateau, a type of geography with a number of consequences. Areas of higher elevations remain glaciated for longer periods, and later population settlement means less time to accumulate variation. Higher elevations may also have been more challenging to colonize, resulting in a lower number of founders and smaller effective population size. In addition, populations in higher altitudes are more likely to be isolated by waterfalls, with net emigration downstream again reducing population size and increasing isolation. Reduced heterozygosity at higher altitudes has been reported in a number of fish species (Trinidadian guppies [*Poeciliareticulata*; Shaw et al. 1991]; eastern mosquitofish, [*Gambusia holbrooki*; Hernandez-Martich and Smith 1990]; brown trout [*Salmo trutta*; Hamilton et al. 1989]) including brook trout in Québec (Angers et al. 1999).

Notably, a significant positive correlation was found between expected heterozygosity and longitude in brook trout populations in west-central and southeastern Labrador. Brook trout populations along the eastern coast of Labrador whose watersheds drain directly to the Atlantic Ocean (Alexis River, Gilbert River, St. Lewis River, St. Mary's River) tend to have higher heterozygosities. Previous studies have shown that anadromous fish have higher levels of heterozygosity than freshwater resident fish (Gyllenstein 1985; Ward et al. 1994; DeWoody and Avise 2000; Tonteri et al. 2007), a trend which is most often attributed to larger long-term effective population sizes. Therefore it is possible that brook trout populations found in watersheds in close proximity to the Atlantic coast are anadromous; a reasonable conjecture given that brook trout are known to have alternate life history styles when found in coastal areas (Curry et al. 2010). In support of this, if brook trout in southeastern Labrador did recolonize the region from an Acadian refugium (as suggested by Danzmann et al. 1998) fish would have had to be euryhaline (a trait associated with anadromy) to disperse back to the mainland. Southeast coast populations would also have been the first recolonized from an Acadian refugium, and hence should be the most variable.

### Contemporary patterns of genetic variation suggest limited ongoing gene flow

Pairwise *F*_ST_ measures in Labrador revealed similar levels of differentiation (mean *F*_ST_= 0.139 among lakes; data not shown) to other studies of brook trout populations, including those in Maine, Québec, and Atlantic Canada (*F*_ST_= 0.099–0.373; Angers and Bernatchez 1998; Hebert et al. 2000; Castric et al. 2001; Castric and Bernatchez 2003). The high level of divergence among populations in Labrador suggests that there are limitations to ongoing gene flow and the hierarchical AMOVA indicates that watershed is the predominant level of differentiation (lakes grouped into watersheds: *F*_CT_= 0.075; watersheds grouped into regions: *F*_SC_= 0.106). Consistent with this, the average pairwise *F*_ST_ among lakes within watersheds was much lower (*F*_ST_= 0.070) than among lakes overall. This pattern can be explained by the freshwater resident lifestyle of the majority of brook trout in Labrador, and is consistent with tagging data indicating that brook trout are moving among lakes (R. Perry, pers. comm.). By way of contrast, a microsatellite and tagging study of brook trout in Indian Bay, insular Newfoundland, revealed very little movement of brook trout among lakes (Adams and Hutchings 2003). The pattern we observed is typical of freshwater fish (Gyllenstein 1985; Currens et al. 1990; Carvalho et al. 1991; Ward et al. 1994; Estoup et al. 1998; Huey et al. 2010) including brook trout (Angers and Bernatchez 1998) where genetic structure is highly influenced by drainage structure. Brook trout may be more mobile in Labrador than insular Newfoundland due to its colder climate and harsher winter—migratory behavior would allow brook trout to maximally exploit resources in this type of environment (Power 2002).

Support for a strong pattern of differentiation among watersheds was provided by the STRUCTURE analysis in which assignment of individuals into *K*= 14 clusters distinguishes the majority of watersheds from one another. One exception to this pattern is that Nikki's Pond (Kenamu River) has a unique cluster assignment, suggesting genetic isolation and differentiation of this lake. This could be due to a natural barrier (e.g., a waterfall), or to the TLH which crosses the Kenamu River watershed along a river that isolates Nikki's Pond from Mercier Lake and Brennan Lake (which are genetically similar to one another). A second exception to the pattern is that the Traverspine River and Eagle River watersheds share a cluster assignment, although each contains a single lake with a unique cluster assignment. The genetic similarity of these two watersheds may be a historical relict reflecting temporal instability of watershed structure; as certain lakes within these separate watersheds are only ∼100 km apart they may have been connected at the time of glacial retreat. Ongoing gene flow of anadromous fish among watersheds is an unlikely explanation, as the river mouths where the watersheds drain are far apart (∼300 km).

We also observed that brook trout populations along the eastern coast of Labrador whose watersheds drain directly into the Atlantic Ocean have STRUCTURE cluster assignments that are similar to one another, but distinct from the rest of Labrador. These are the same watersheds (Alexis River, Gilbert River, St. Lewis River, St. Mary's River) that tend to have higher levels of heterozygosity and are hypothesized to contain anadromous brook trout. The connectedness of these coastal populations, also supported by low levels of differentiation (pairwise *F*_ST_= 0.066) and hence membership in the same neighbor-joining dendrogram cluster (Fig. 3), provides further evidence of an anadromous life history.

### Implications for management of brook trout in southeastern Labrador

Previous studies have reported extensive loss of brook trout from their native habitat due to land development and habitat alteration (Hudy et al. 2008; Stranko et al. 2008; Kanno et al. 2010; Waco and Taylor 2010). The populations surveyed in this study are under the same anthropogenic threats due to the proximity of the TLH which crossed 20 watersheds in its Phase II section (Red Bay to Cartwright) alone (Jacques Whitford Environment Limited 1998). Therefore it is important to collect baseline data on the genetic diversity and structure of populations to make management recommendations and monitor populations in the future. Previous management decisions made by the Wildlife Division of the Newfoundland and Labrador Department of Environment and conservation relied on Adams and Hutchings (2003) which found that brook trout do not migrate among lakes. By way of contrast, microsatellite data combined with preliminary tagging data (Robert Perry, pers. comm.) suggest that brook trout in Labrador do move among lakes within watersheds, and that watersheds represent the unit of reproductive isolation. Hence it may be more appropriate to direct management decisions at the watershed level in Labrador.

Gibson et al. (2005) reported that 53% of the culverts assessed along Phase II of the TLH are not suitable for fish passage due to either poor design or installation. This causes concern with respect to loss of spawning and rearing habitats, as well the introduction of barriers to migration. Barriers to migration are especially worrisome in light of the microsatellite evidence presented here that brook trout in Labrador are in fact moving among lakes. Fortunately, the Gibson et al. (2005) study led to the improvement of a number of culverts along Phase II, as well as the revision and development of better stream crossings for Phase III. Regardless, it is important to continue to monitor populations, as easier access to remote watersheds can also lead to fisheries’ exploitation.

### Evidence for dual routes of post-Wisconsinan colonization

The pattern of microsatellite diversity in Labrador brook trout is clearly influenced by contemporary factors, in particular watershed structure. However, a role for historical events is also evident, as despite structure at the level of watershed, certain watersheds are genetically similar, suggesting they were colonized by the same founding population, and there is little genetic differentiation among regions. The isolation-by-distance analysis also indicated that historical waterway distances have been more influential than contemporary ones in shaping the present-day genetic structure of brook trout populations in northeastern Canada. Consistent with this, brook trout populations in the northernmost portion of their range are evolutionarily young and likely not yet in equilibrium between migration and genetic drift. Current patterns of microsatellite variability therefore reflect colonization history overlaid with developing divergence due to ongoing limitations to gene flow.

Microsatellites have been successfully utilized to infer colonization patterns (Angers and Bernatchez 1998; Taylor and McPhail 2000; Lu et al. 2001; Koskinen et al. 2002), and can reveal patterns that are undetectable with mtDNA or other phylogeographic markers (Flanders et al. 2009). This is the case for Labrador brook trout; only one mtDNA haplotype was found during a survey of genetic variation of freshwater fish in Labrador (S. Carr, unpubl. report) and Danzmann et al. (1998) reported the predominance of a single mtDNA haplotype in the entire northern portion of the range.

Here we detected evidence for *K*= 2 genetic groups from the STRUCTURE analysis in which we applied the Evanno et al. (2005) ad hoc algorithm; one that includes lakes in northern and west-central Labrador, and another encompassing lakes in all other regions. This deep level of genetic differentiation suggests that northern and west-central Labrador populations of brook trout may have been colonized from a different refugial source than the rest of the northeastern range.

Black et al. (1986) proposed that brook trout from the Atlantic and Mississippi refugia dispersed through Québec then into Labrador, while other fish from the Atlantic refugium invaded coastal regions. Alternatively, Danzmann et al. (1998) suggested that brook trout in the northeastern parts of their native range may have originated from a more northeasterly Acadian refugium. Our results provide support for a combination of the two hypotheses; northern and west-central Labrador may have been founded by some combination of fish from the Atlantic and/or Mississippian refugia, while southeastern populations were founded by those from an Acadian refugium. In support of this, the neighbor-joining dendrogram clusters northern Labrador populations with those in west-central Labrador (except Atikonak Lake). A third hypothesis is that northern and west-central Labrador lakes were colonized from a northern coastal refugium/nunatak located in the Torngat Mountains in northern Labrador (Pielou 1991). In this scenario central Labrador lakes would have been colonized subsequent to northern ones, accounting for their reduced diversity. Evidence for a Labrador refuge is also supported by the phylogeographic origins of Arctic charr (Wilson et al. 1996), as fish in this region were not represented by the Laurentian lineage which colonized Quebec, New Brunswick, and New England.

## Conclusions

We note that microsatellite variation is sufficient to detect the presence of two refugial sources of brook trout in Labrador, whereas previous mtDNA studies revealed little to no variation (S. Carr, pers. comm.; Danzmann et al. 1998). In addition to the strong role of the historical process of recolonization on shaping the pattern of present-day genetic structure of brook trout populations in Labrador, we find evidence that a contemporary factor, watershed structure, is also important. Wildlife management practices relying on the data from Newfoundland brook trout have assumed that there is no movement of fish among lakes in Labrador; we found evidence in contradiction of this which has important implications for future management decisions with respect to the recent opening of the TLH. We point to the importance of documenting the genetic structure of a species across its entire range rather than extending conclusions from a portion of the range, especially in species like brook trout that are ecologically complex and display a variety of life history traits.
